# A miR-146a-5p/TRAF6/NF-kB p65 axis regulates pancreatic cancer chemoresistance: functional validation and clinical significance

**DOI:** 10.7150/thno.40566

**Published:** 2020-03-04

**Authors:** Qingcai Meng, Chen Liang, Jie Hua, Bo Zhang, Jiang Liu, Yiyin Zhang, Miaoyan Wei, Xianjun Yu, Jin Xu, Si Shi

**Affiliations:** 1 Department of Pancreatic Surgery, Fudan University Shanghai Cancer Center, Shanghai 200032, China.; 2 Department of Oncology, Shanghai Medical College, Fudan University, Shanghai 200032, China.; 3 Shanghai Pancreatic Cancer Institute, Shanghai 200032, China.; 4 Pancreatic Cancer Institute, Fudan University, Shanghai 200032, China.

**Keywords:** microRNA, pancreatic cancer, chemoresistance, prognosis

## Abstract

**Background**: Dysregulated microRNA (miRNA) expression in cancer can act as a key factor that modifies biological processes, including chemoresistance. Our study aimed to identify the miRNAs associated with gemcitabine (GEM) resistance in pancreatic ductal adenocarcinoma (PDAC) and to explore the potential mechanisms.

**Methods**: The miRNA microarray was used to identify miRNAs associated with GEM resistance. Quantitative real-time PCR was used to examine miR-146a-5p expression in paired PDAC and adjacent normal tissues. Bioinformatics analysis, luciferase reporter assays, and chromatin immunoprecipitation assays were used to confirm tumor necrosis factor receptor-associated factor 6 (TRAF6) as a direct target of miR-146a-5p and to explore the potential transcription factor binding and regulation by miR-146a-5p. *In vitro* and *in vivo* experiments were performed to investigate the mechanisms.

**Results**: MiR-146a-5p expression was significantly decreased in PDAC tissues compared with adjacent normal tissues, and miR-146a-5p expression correlated with prognosis in PDAC patients. Functional studies indicated that miR-146a-5p suppressed PDAC cell proliferation and sensitized PDAC cells to GEM chemotherapy by targeting the 3'-untranslated region (3′-UTR) of TRAF6. MiR-146a-5p was also observed to downregulate the TRAF6/NF-κB p65/P-gp axis, which regulates PDAC cell growth and chemoresistance.

**Conclusions**: Taken together, the results indicate that the miR-146a-5p/TRAF6/NF-κB p65 axis drives pancreatic chemoresistance by regulating P-gp, suggesting that miR-146a-5p may be utilized as a new therapeutic target and prognostic marker in PDAC patients.

## Introduction

As the most fatal gastrointestinal malignancy, pancreatic ductal adenocarcinoma (PDAC) represents the fourth highest cause of cancer-related death worldwide, with a 5-year survival rate of only 8% 1,2. Gemcitabine (GEM)-based chemotherapy regimens constitute an important component of multimodal cancer treatment, but chemotherapeutic resistance is a major challenge for the management of PDAC 3,4. The molecular mechanisms underlying chemoresistance in different types of cancer include drug inactivation and target alteration, epithelial-mesenchymal transition, DNA damage repair, cell death inhibition, inherent cell heterogeneity, and epigenetic effects 5. Thus, a comprehensive understanding of the mechanisms governing chemoresistance in PDAC may provide new therapeutic strategies and better drug selection for improving the prognosis of this lethal disease.

MicroRNAs (miRNAs) are a class of evolutionarily conserved small noncoding RNAs 19~25 nucleotides in length that negatively regulate genes at the posttranscriptional level by binding to the 3′-untranslated region (3′-UTR) of target mRNAs 6,7. Increasing evidence has demonstrated that miRNAs act as key factors in various kinds of tumors to modify biological processes, such as cell proliferation, differentiation, metabolism, apoptosis, and chemoresistance 8-15. Here, we established pancreatic cancer cell lines with acquired chemoresistance to GEM (GR cells). Moreover, the miRNA microarray study showed that miR-146a-5p was significantly downregulated in GR cells compared with parental cells, suggesting its potential role in modulating chemoresistance.

Tumor necrosis factor receptor-associated factor 6 (TRAF6), a member of the TRAF family, has been previously identified as a signal transducer in the regulation of inflammation and immunity 16-18. TRAF6 could function as a key activator of NF-κB signaling by binding to other signaling molecules, including NF-κB-inducing kinase 19,20. NF-κB signaling has been shown to induce acquired resistance against GEM in the treatment of PDAC in multiple ways 21. Studies have demonstrated that crosstalk of NF-κB with other regulators, such as SOCS3, TRIM31, ITCH, and TNIP1, leads to GEM resistance 22-24. Additionally, NF-κB was shown to bind the promoter regions of P-glycoprotein (P-gp), which was reported to contribute to GEM resistance 25. Recent studies have indicated that TRAF6 regulates tumorigenesis by inhibiting apoptosis and stimulating proliferation and invasion in various cancers, including PDAC 26-29. However, whether TRAF functions to confer resistance to GEM by regulating the NF-κB/P-gp axis remains unknown.

Herein, we confirmed that TRAF6 is a direct target of miR-146a-5p and is also upregulated in PDAC-GR cells. Next, we demonstrated that miR-146a-5p regulates the carcinogenesis and chemoresistance of PDAC cells by repressing TRAF6 expression *in vitro* and *in vivo*. Furthermore, survival analysis revealed that lower miR-146a-5p expression and high TRAF6 expression levels were both correlated with poor prognosis in PDAC patients. We also found that the miR-146a-5p/TRAF6/NF-κB p65 axis was involved in regulating PDAC cell growth and chemoresistance. Taken together, our data points towards the efficacy of systemic miR-146a-5p delivery as a potential targeted therapy.

## Materials and methods

### Cell lines and reagents

The human PDAC cell lines Capan-1, MiaPaCa-2, BxPC-3, SW1990, and PANC-1 were obtained from the American Type Culture Collection (ATCC). The culture conditions for all cells have been described previously 30, 31. Human pancreatic ductal epithelial (HPDE) cells were grown in complete keratinocyte serum-free medium containing bovine pituitary extract (50 µg/mL) and epidermal growth factor (5 ng/mL) (Gibco, Carlsbad, CA, USA). All cells were cultured in a humidified atmosphere of 5% CO_2_ at 37 °C. GEM-resistant MiaPaCa-2 cells (GR cells) were obtained by researchers in our laboratory by gradually increasing the GEM doses. GEM (1000 ng/mL) was added into the medium to maintain the resistant MiaPaCa-2-GEM cell phenotype for a long time. GEM was obtained from Selleck (Houston, TX, USA).

### Tissue specimens

Tissue microarrays (TMAs) were obtained from 87 PDAC patients who underwent surgical resection at Fudan University Shanghai Cancer Center (FUSCC) from 2010 to 2012. Two independent pathologists conducted the strict pathological diagnoses and stages independently. Strict postoperative follow-ups were performed for all patients. All procedures were performed after obtaining approval from the Clinical Research Ethics Committee of FUSCC, and informed consent was obtained from each patient.

### Cell transfection

Hsa-miR-146a-5p mimics (sense: 5′-UGAGAACUGAAUUCCAUGGGUU-3′; antisense: 5′-AACCCAUGGAAUUCAGUUCUCA-3′) and a nonspecific mimic control (NC, sense: 5′-UUUGUACUACACAAAAGUACUG-3′; antisense: 5′-CAGUACUUUUGUGUAGUACAAA-3′), hsa-miR-146a-5p inhibitors (sense: 5′-AACCCAUGGAAUUCAGUUCUCA-3′) and the NC (sense: 5′-CAGUACUUUUGUGUAGUACAAA-3′) were synthesized by RiboBio (Guangzhou, China). PANC-1 and SW1990 cells were transfected with miR-146a-5p mimics, miR-146a-5p inhibitors, or their NC using Lipofectamine 2000 (Invitrogen, Carlsbad, CA, USA) according to the manufacturer's instructions. TRAF6-knockdown and overexpression plasmids were constructed by Shanghai GeneChem (Shanghai, China). Plasmid transfection was performed using Lipofectamine 2000 according to the manufacturer's instructions.

### RNA isolation and quantitative real-time PCR (qRT-PCR)

Total RNA was extracted from cells and tumor samples with TRIzol Reagent (Invitrogen, Carlsbad, CA, USA) and was subsequently reverse transcribed into cDNA using a PrimeScript RT Reagent Kit (TaKaRa, Shanghai, China) according to the manufacturer's instructions. Ninety-three paired PDAC samples originated from patients who were histopathologically and clinically diagnosed at FUSCC. The expression of candidate genes was determined by qRT-PCR using an ABI 7900HT Real-Time PCR System (Applied Biosystems, USA). The 2^-ΔΔCt^ method was used to calculate changes in the mRNA expression levels. The expression values of miRNAs and mRNAs were normalized against the endogenous controls U6 and β-actin. The primers were purchased from RiboBio (Guangzhou, China), and the sequences are listed in Table S1.

### MiRNA sequencing analysis

Total RNA was isolated from the parental MiaPaCa-2 cells and MiaPaCa-2-GR cells using TRIzol Reagent. The TruSeq Small RNA Library Preparation Kit (Illumina, San Diego, CA, USA) was used for library preparation. Sequencing was performed using an Illumina HiSeq 2000 sequencing system, and 10 Mb of clean reads was analyzed using routine algorithms (KangChen Biotech, Shanghai, China).

### Western blot analysis

Total proteins were extracted using cell lysates in RIPA buffer, and the protein concentration was measured using a BCA Protein Assay Kit (Thermo Fisher Scientific, Waltham, MA, USA). Western blots were performed as previously described 32. Antibodies against TRAF6 and β-actin were purchased from Proteintech. The primary rabbit anti-P-glycoprotein (P-gp) and anti-NF-κB p65 antibodies were purchased from Cell Signaling Technology.

### Immunohistochemical (IHC) staining

IHC staining with antibodies against TRAF6 (Proteintech, Chicago, IL, USA), P-gp (Cell Signaling Technology, Danvers, MA, USA) and NF-κB p65 (CST, Danvers, MA, USA) was performed and scored to determine the protein expression according to standard procedures as previously described 30. The protein expression levels were calculated by multiplying the positivity and intensity scores: 0, negative; 1-3, weakly positive; 4-6, moderately positive and > 6, strongly positive. Then, we divided the patients into two groups (0-3, low expression and ≥ 4, high expression) and performed survival analyses.

### Cell viability and cell cytotoxicity assays

Cell viability and cell growth inhibition were determined as previously described 31,33. Briefly, cells were plated in 96-well plates at a density of 2000 cells per well, and CCK-8 (Gaithersburg, MD, USA) was added at the end of the experiment. The concentration of GEM that inhibited 50% of the cell viability was extrapolated from a nonlinear least squares curve that fit the dose-response curves (GraphPad Software Inc, La Jolla, CA, USA) and was then used to obtain the IC_50_ values.

### Colony formation assay

Cells were seeded into 6-well plates at an initial density of 500 cells/well and then incubated for 2 weeks. The colonies were fixed in 4% paraformaldehyde and stained with 0.1% crystal violet (Sigma, St. Louis, MO, USA). The visible colonies were counted using light microscopy. Triplicate wells were measured for each treatment group.

### Flow cytometry

Cell apoptosis was determined by using an Annexin V-PE Apoptosis Detection Kit (BD, La Jolla, CA, USA) according to the manufacturer's instructions. In brief, cells were pelleted by centrifugation and then resuspended in 100 μL of annexin V binding buffer. After incubation, the cells were washed and subjected to a FACSCalibur flow cytometer. The data were analyzed by FlowJo software (FlowJo LLC, Ashland, OR, USA).

### Tumorigenesis study

BALB/c-nu mice (6 weeks of age, Shanghai SLAC Laboratory Animal Co, Ltd., China) were housed under specific pathogen-free conditions. A total of 3 × 10^6^ cells in 100 µl of PBS were subcutaneously inoculated into the left flank of each mouse. From the time of the formation of palpable tumors, miR-146a-5p agomir and agomir NC (RiboBio, Guangzhou, China) were injected into the tumor at multiple sites twice per week. Each group was randomly divided into two subgroups and subjected to intraperitoneal injection of GEM (20 mg/kg) or PBS (100 μL; negative control) twice per week. The tumor size was measured using an external caliper twice per week and calculated by the formula V = (length × width^2^)/2. The mice were euthanized at the 5th week, and the tumors were surgically dissected for IHC examination. The study was performed in strict accordance to the guidelines of the Committee on the Ethics of Animal Experiments of Fudan University.

### Dual-luciferase reporter assay

The mutant 3′-UTR of TRAF6, which contained a point-mutated sequence in the binding region of miR-146a-5p, was generated by using a site-directed mutagenesis kit from Fast Mutagenesis System (RiboBio, Guangzhou, China). PANC-1 and SW1990 cells were plated on 96-well culture plates and transfected with the reporter construct with the Renilla luciferase expression vector pRL-TK (Promega, Madison, WI, USA) using Lipofectamine 2000 (Invitrogen, Gaithersburg, MD, USA). After transfection for 48 h, the cells were assayed for luciferase activities using a dual-luciferase system (Promega, Madison, WI, USA), as previously described 34.

### Bioinformatics study

Five software programs, including TargetScan (http://www.targetscan.org/vert_71/), PicTar (http://pictar.mdc-berlin.de/cgi-bin/new_PicTar_vertebrate.cgi), miRanda (http://www.microrna.org/microrna/home.do), RNA22 (https://cm.jefferson.edu/rna22/) and PITA (https://genie.weizmann.ac.il/pubs/mir07/mir07_prediction.html), were used to predict the biological targets of miR-146a-5p.

### Public datasets analysis

The Cancer Genome Atlas (TCGA)-PAAD containing the RNA expression data (Level 3) of pancreatic cancer patients analyzed by RNA-seq by expectation-maximization was downloaded from the Cancer Genomics Brower of the University of California, Santa Cruz (UCSC; https://genome-cancer.ucsc.edu/). In total, 178 primary pancreatic cancer samples from patients with detailed expression data were chosen from the updated TCGA database according to the parameters mentioned above.

### Statistics

Data are presented as the means ± SD. Independent Student's t-test or one-way ANOVA and Tukey's post hoc test were used to evaluate the data. Spearman correlation analysis was used to determine the association of miR-146a-5p expression with TRAF6 expression. The correlation between miR-146a-5p and clinicopathological characteristics was assessed using χ^2^-test. Kaplan-Meier analyses log-rank test were used to determine survival analysis. All statistical analyses were performed using SPSS 19.0 software (IBM, Chicago, IL, USA).* P*-values less than 0.05 were considered statistically significant.

## Results

### MiR-146a-5p is aberrantly downregulated in GEM-resistant PDAC cells and correlated with poor prognosis for PDAC patients

**Figure 1:**
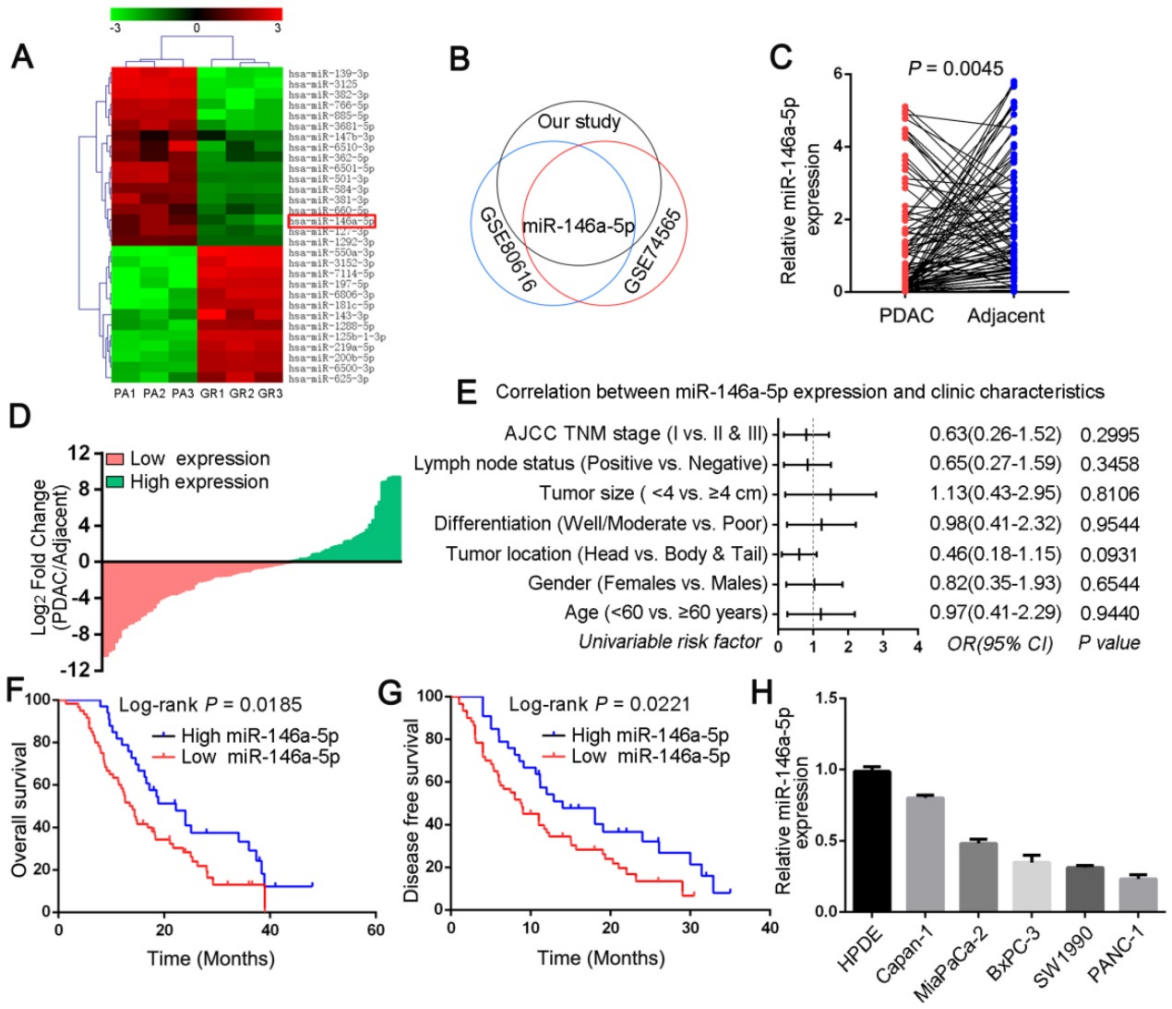
The expression and clinical significance of miR-146a-5p in pancreatic ductal adenocarcinoma (PDAC). (A) Comparison of microRNA (miRNA) expression in MiaPaCa-2 parental and GR cells by using a miRNA microarray. Each cell was tested in triplicate. (B) The overlapping miRNAs associated with PDAC-GR cells from three different studies (our study, GSE74565 and GSE80616) are shown in a Venn diagram. (C) Expression of miR-146a-5p in tumor and adjacent normal tissues from a cohort of 93 PDAC patients was determined by qPCR and normalized against endogenous U6 expression. (D) Overexpression of miR-146a-5p was frequent in tumor samples from PDAC patients (36.5%, 34 of 93 patients). (E) Correlations of miR-146a-5p levels in PDAC tissues and clinicopathological features of PDAC. Statistical significance was determined by the χ^2^-test. (F-G) Kaplan-Meier analysis indicated that downregulation of miR-146a-5p was significantly associated with worse prognosis in 93 PDAC patients, with shorter overall survival (OS, p = 0.0185) and disease-free survival (DFS; p = 0.0221). (H) qPCR analysis of miR-146a-5p expression in the indicated human pancreatic cancer cell lines and the HPDE cell line.

To investigate candidate regulators of GEM chemoresistance in PDAC, we first generated PDAC cell line models with acquired GEM resistance (MiaPaCa-2-GR and SW1990-GR cells) by culturing parental PDAC cells (PA cells) with increasing concentrations of GEM over a period of approximately 6 months (Figure S1A). The inhibitory concentration (IC_50_) of GEM in the GR cells was determined via CCK-8 cytotoxicity assays, which revealed that the GR cells were more resistant to GEM than were the PA cells (Figure S1B-C). Next, to screen and identify the miRNAs that can enhance the response of PDAC cells to GEM chemotherapy, a miRNA microarray was used to profile the simultaneously upregulated or downregulated miRNAs between MiaPaCa-2-GR cells and their respective parental cells (Figure 1A). Combined with two other miRNA microarrays from the Gene Expression Omnibus (GEO) database (GSE74565 and GSE80616), we observed that miR-146a-5p was significantly downregulated in all PDAC-GR cell lines (Figure 1B). In addition, our qRT-PCR data showed low miR-146a-5p expression in the GR cell lines compared with that in the corresponding parental cells (Figure S1D), suggesting a potential role for miR-146a-5p in regulating chemoresistance. Moreover, to determine the clinical significance of miR-146a-5p in PDAC, we assessed its expression in a cohort comprising 93 pairs of PDAC tumors and matched adjacent normal tissues. Indeed, compared with the adjacent tissues, PDAC tumor tissues had lower miR-146a-5p expression (Figure 1C-D). As shown in Figure 1E, there was no obvious relationship between miR-146a-5p expression and clinicopathological features, such as age, sex, tumor size, positive lymph nodes, and TNM stage (Table S2). More importantly, Kaplan-Meier analysis indicated that miR-146a-5p downregulation was significantly associated with a worse prognosis with shorter overall survival (OS,* p* = 0.0185; Figure 1F) and disease-free survival (DFS; *p* = 0.0221; Figure 1G), and Cox regression analysis showed that miR-146a-5p could be a prognostic marker to predict the outcomes of PDAC patients (Table S3). In addition, our results revealed downregulated miR-146a-5p expression in different PDAC cell lines compared with the expression in HPDE cells (Figure 1H).

### MiR-146a-5p inhibits the proliferation and chemoresistance of PDAC cells

**Figure 2:**
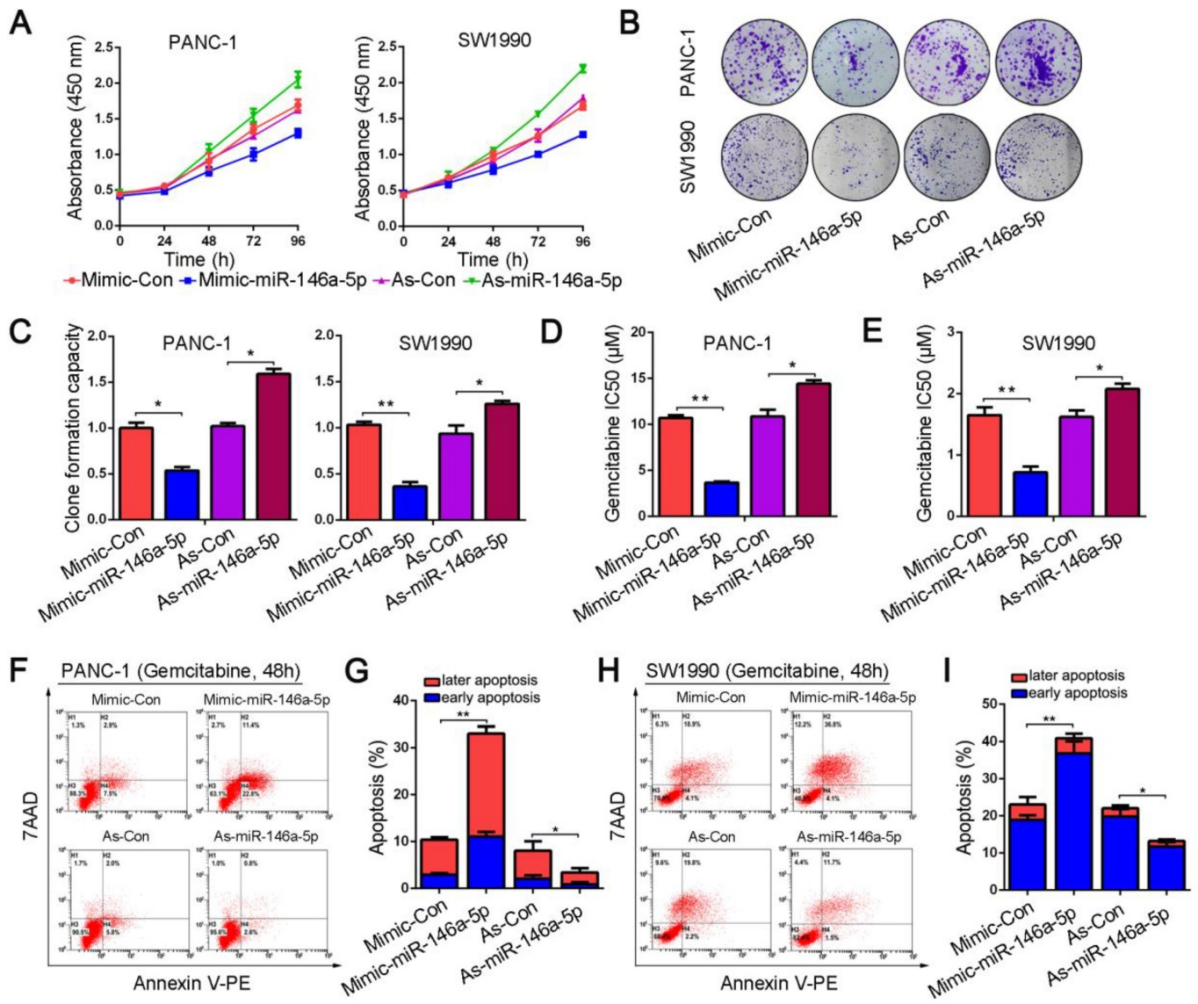
MiR-146a-5p inhibits the proliferation and chemoresistance of PDAC cells. (A) Cell proliferation in PANC-1 and SW1990 cells transfected with miR-146a-5p mimics, inhibitors or their respective negative controls (NCs) was detected by the CCK-8 assay. (B-C) Representative images and statistical analysis of colony formation in PANC-1 and SW1990 cells transfected with miR-146a-5p mimics, inhibitors or their respective NCs (*P < 0.05, **P < 0.01). (D-E) IC_50_ value of gemcitabine (GEM) measured in PANC-1 and SW1990 cells transfected with miR-146a-5p mimics, inhibitors or their NCs (*P < 0.05, **P < 0.01). (F-I) The apoptosis rate of PANC-1 and SW1990 cells transfected with miR-146a-5p mimics, inhibitors or their NCs and treated with GEM (10 µM, 48 h) was measured by flow cytometric analysis using annexin V/7-AAD staining (*P < 0.05, **P < 0.01).

To investigate the potential function of miR-146a-5p, we transfected mimics and inhibitors of miR-146a-5p into PANC-1 and SW1990 cell lines. The results from the CCK-8 and colony formation assays showed that ectopic expression of miR-146a-5p significantly inhibited the proliferation and colony-forming abilities of the cells (Figure 2A-C). Next, we examined the role of miR-146a-5p on GEM chemoresistance in PDAC cells. PANC-1 and SW1990 cells were treated with GEM at various concentrations for 48 h, and viability was measured by the CCK-8 assay. We observed that the IC_50_ values of GEM were remarkably reduced in PDAC cells transfected with the miR-146a-5p mimic, suggesting that miR-146a-5p enhanced the cytotoxicity of GEM (Figure 2D-E). Conversely, inhibition of miR-146a-5p expression markedly increased the GEM chemoresistance of PDAC cells, with obviously increased IC_50_ values. As apoptosis induction is a key mechanism mediating the antitumor effect of GEM, we also explored the effect of miR-146a-5p on apoptosis by performing flow cytometry analysis of annexin V-stained cells. After treatment with GEM for 48 h, similar results were obtained, showing that the GEM-induced apoptosis rate greatly increased in miR-146a-5p-mimic cells, whereas downregulation of miR-146a-5p inhibited apoptosis after GEM treatment (Figure 2F-I). Taken together, these results indicate that miR-146a-5p not only inhibits cell proliferation but also enhances the cytotoxicity of GEM.

### MiR-146a-5p sensitizes chemotherapeutic efficacy in pancreatic xenograft tumors

**Figure 3:**
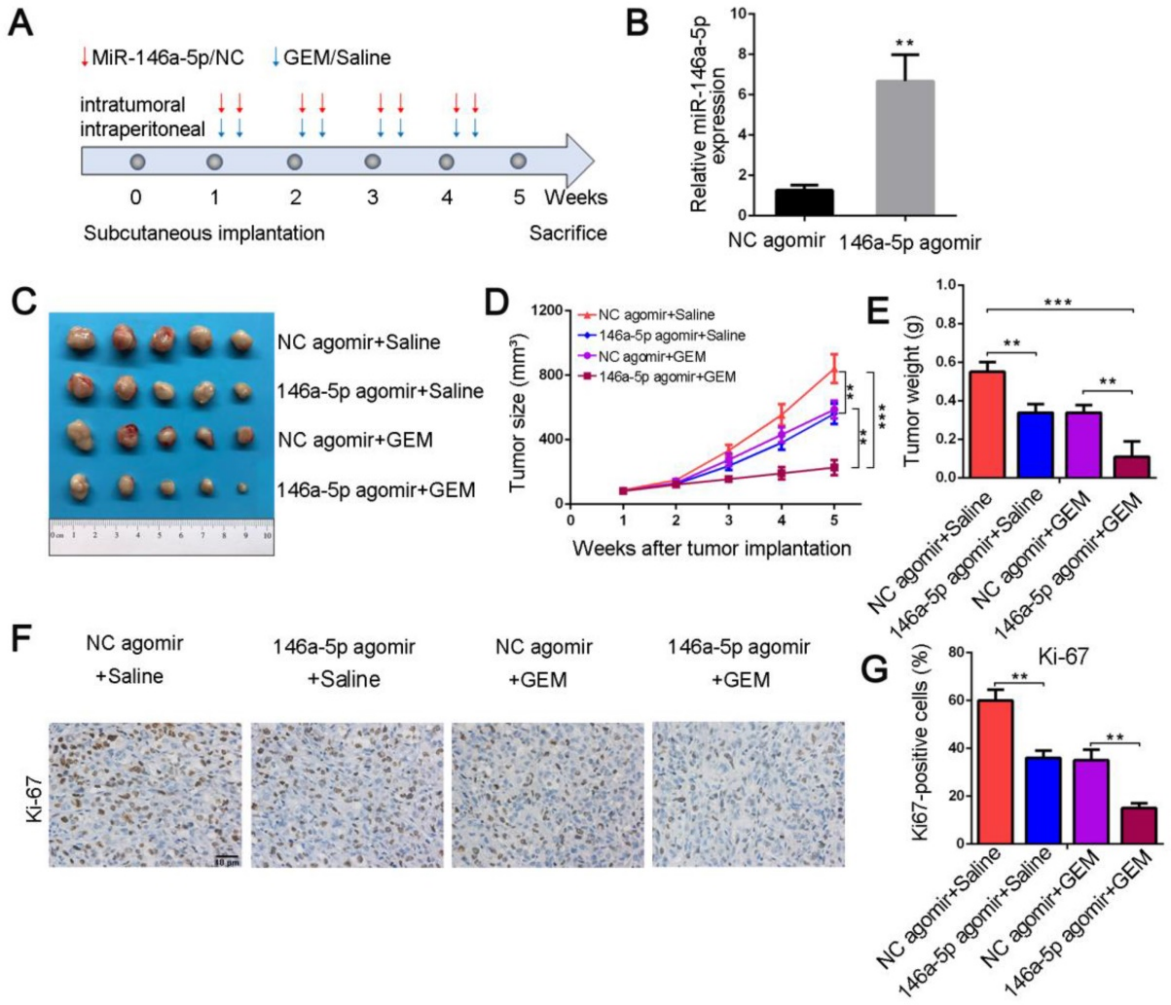
MiR-146a-5p sensitizes chemotherapeutic efficacy *in vivo*. (A) Schematic outline of the combination therapy in a pancreatic xenograft tumor model. (B) MiR-146a-5p expression in transplanted tumors was detected by qPCR (**P < 0.01). (C) Representative images showing the tumors formed in the four treatment groups (n = 5 for each group). (D) Tumor growth curves were drawn according to the measured tumor volumes (**P < 0.01, ***P < 0.001). (E) Tumor weights of the four groups were measured at the 5th week after subcutaneous transplantation (**P < 0.01, ***P < 0.001). (F-G) Representative tumor tissue sections of the xenografts from the four groups were analyzed for the proliferation marker Ki-67 using immunohistochemistry, and the percentages of Ki67-positive cells were measured (scale bar, 40 µm, **P < 0.01).

To further confirm whether miR-146a-5p correlated with GEM resistance *in vivo*, we established PDAC xenograft models. When transplanted mice presented palpable xenografted tumors, they were treated with miR-146a-5p and GEM twice per week (Figure 3A). The expression level of miR-146a-5p in xenograft tumor tissues treated with miR-146a-5p agomir was increased compared with that in tissues treated with negative control (NC) agomir (Figure 3B). As shown in Figure 3C-E and Figure S2A-C, in the absence of GEM treatment, the tumor sizes and weights were significantly lower in the miR-146a-5p agomir-treated group than in the NC agomir group. Moreover, the tumor sizes and weights were further reduced in the miR-146a-5p agomir with GEM treatment group. Subsequent IHC examination of the expression levels of the proliferation marker Ki-67 demonstrated that cell proliferation was inhibited and apoptosis was significantly increased in the miR-146a-5p agomir group with GEM treatment (Figure 3F-G and Figure 2SD-E). Collectively, these results demonstrated that miR-146a-5p could inhibit tumor growth and sensitize PDAC cells to GEM treatment.

### MiR-146a-5p targets the 3′-UTR of TRAF6 and suppresses its expression

**Figure 4:**
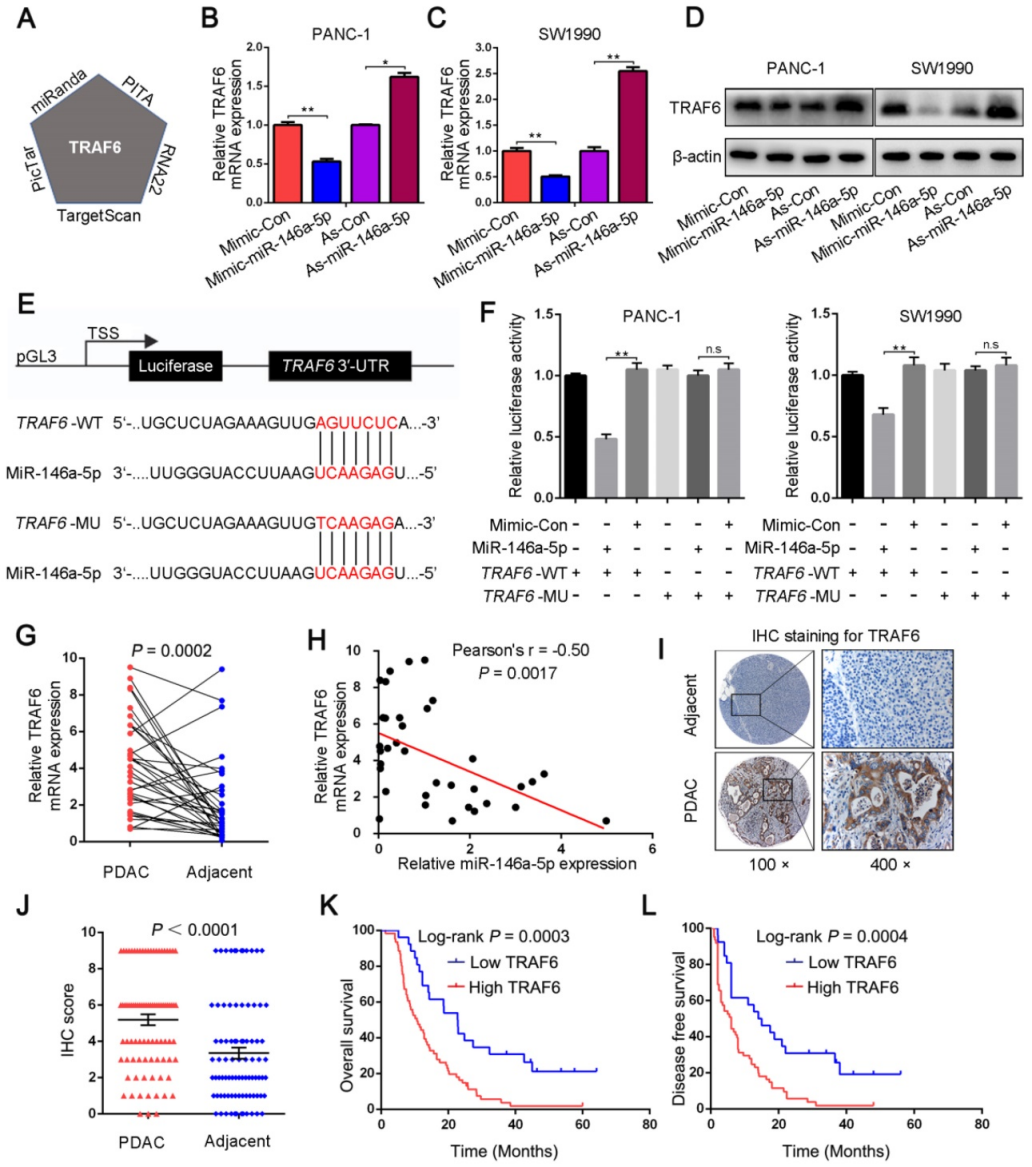
MiR-146a-5p targets the 3′-UTR of TRAF6 and suppresses its expression. (A) TRAF6 was identified as the putative target of miR-146a-5p in five miRNA target prediction algorithms. (B-D) qPCR and Western blot assays were used to detect TRAF6 mRNA and protein levels in PDAC cells transfected with miR-146a-5p mimics, inhibitors or their NCs. (E) A schematic showing the sequence alignment of wild-type (WT) and mutant (MU) miR-146a-5p target sites on the 3′-UTR of TRAF6. Both the WT and MU 3'-UTRs were cloned into luciferase reporter constructs. (F) Dual-luciferase analysis of cotransfection of miR-146a-5p with the WT or MU 3′-UTR of TRAF6 in PDAC cells (n.s, not significant, **P < 0.01). (G) TRAF6 expression in tumor and adjacent normal tissues from 36 PDAC patients was determined by qPCR. (H) The relationship of the expression between miR-146a-5p and TRAF6 in 36 PDAC tissues (*P* = 0.0017). (I-J) Tissue microarray (TMA) analysis by IHC staining showed that increased TRAF6 protein expression was observed in 87 PDAC tissues (*P* < 0.0001). (K-L) Kaplan-Meier analysis showed that increased TRAF6 expression was significantly associated with poorer OS and DFS in 87 PDAC patients.

We next investigated the mechanism by which miR-146a-5p regulates cell proliferation and GEM resistance in PDAC cells. First, the potential target genes of miR-146a-5p were explored through bioinformatics analysis using five different databases: TargetScan, PITA, RNA22, PicTar, and miRanda. TRAF6 was identified as a potential target of miR-146a-5p (Figure 4A and Table S4); therefore, we then analyzed the mRNA and protein expression of TRAF6 in response to changes in miR-146a-5p expression. The results showed that TRAF6 expression was reduced in cells treated with the miR-146a-5p mimic and increased in cells treated with the miR-146a-5p inhibitor (Figure 4B-D). To further investigate the significance of the interaction between miR-146a-5p and TRAF6, a dual-luciferase reporter assay was performed. Then, the predicted miR-146a-5p binding site on the 3′-UTR of wild-type TRAF6 (WT, 5′-GAGGCCGG-3′) was mutated (MU, 5′-CACCCGCC-3′), and the full-length 3'-UTR was cloned into a luciferase reporter vector (Figure 4E). After the vector containing the mutant 3'-UTR was transfected into PANC-1 and SW1990 cells, the inhibitory effect of miR-146a-5p on the luciferase activity was decreased (Figure 4F), confirming that miR-146a-5p can directly bind to TRAF6 and suppress its expression. Moreover, the expression levels of TRAF6 were determined in 36 pairs of PDAC tumors and adjacent normal tissues. The results revealed that significantly higher TRAF6 expression was observed in PDAC tissues than in normal tissues (Figure 4G), and an inverse correlation between miR-146a-5p and TRAF6 was detected (Figure 4H). Similarly, both TCGA and genotype-tissue expression project (GTEx) databases showed increased TRAF6 expression in PDAC tissues compared with that in normal tissues (Figure S3A). Consistent with the above results, the TMA analysis by IHC staining showed that increased TRAF6 protein expression was observed in 87 PDAC tissues (Figure 4I-J) and correlated with more positive lymph nodes, poor differentiation and a more severe TNM stage (Figure S3B-C and Table S5). Furthermore, increased TRAF6 expression was significantly associated with a worse prognosis, with poorer OS and DFS (Figure 4K-L).

### TRAF6 mediates the effect of miR-146a-5p on GEM resistance in PDAC cells

**Figure 5:**
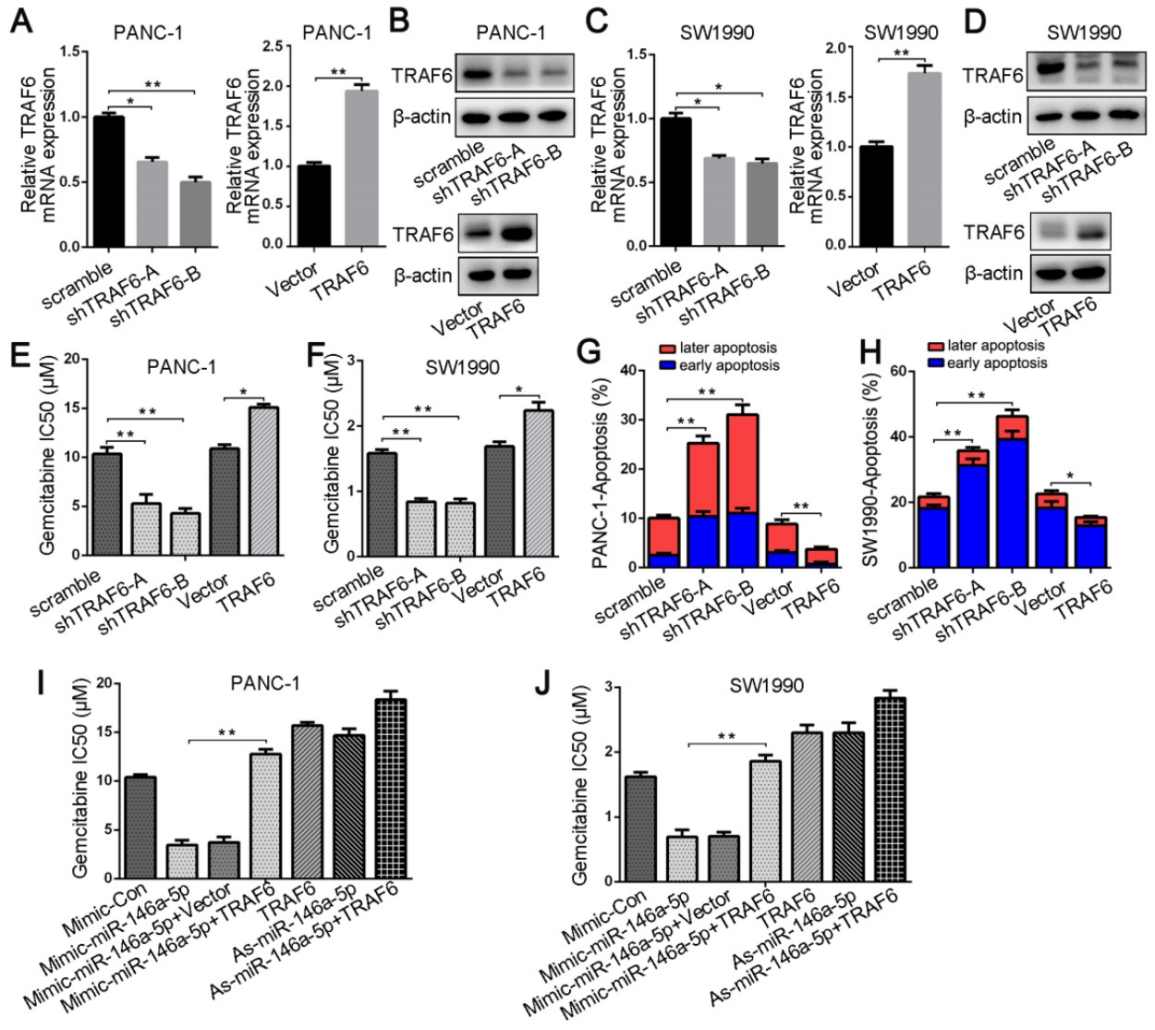
TRAF6 mediates the effect of miR-146a-5p on GEM resistance in PDAC cells. (A-D) qPCR and Western blotting analyses confirmed TRAF6 knockdown and overexpression in PANC-1 and SW1990 cells. (E-F) The IC_50_ values of GEM in PANC-1 and SW1990 cells transfected with the TRAF6-specific shRNA or TRAF6 overexpression vector (*P < 0.05, **P < 0.01). (G-H) The apoptosis rate of PANC-1 and SW1990 cells transfected with TRAF6 shRNA or overexpression vector and treated with GEM (10 µM, 48 h) was measured (*P < 0.05, **P < 0.01). (I-J) Comparison of the IC_50_ values of GEM in PANC-1 and SW1990 cells treated with miR-146a-5p mimics/inhibitor either alone or in conjunction with transfection of the TRAF6-overexpressing vector (*P < 0.05, **P < 0.01).

To investigate the relationship between TRAF6 expression and GEM resistance in PDAC cells, we first transfected either TRAF6-specific shRNA or a vector overexpressing TRAF6; into PANC-1 and SW1990 cells. qPCR and Western blotting analyses confirmed TRAF6 knockdown and overexpression, respectively (Figure 5A-D). Silencing TRAF6 dramatically decreased the IC_50_ values of GEM and increased its cytotoxic effect in PDAC cells, whereas TRAF6 overexpression increased the IC_50_ value (Figure 5E-F). Apoptotic cells analyzed by flow cytometry also confirmed that the GEM-induced apoptosis rate in TRAF6-knockdown cells was increased, whereas TRAF6 overexpression decreased the apoptosis rate (Figure 5G-H). In addition, to further verify whether TRAF6 mediates the effect of miR-146a-5p on GEM chemoresistance, we cotransfected both the miR-146a-5p mimic and TRAF6 overexpression vector into PDAC cells. The results revealed that the lower IC_50_ values induced by the miR-146a-5p mimic were reversed upon TRAF6 overexpression, and the higher IC_50_ values in cells with miR-146a-5p inhibitor were further increased when TRAF6 was overexpressed (Figure 5I-J), suggesting that the effects of miR-146a-5p on the potency of GEM were inhibited by TRAF6 overexpression. Therefore, these results demonstrate that miR-146a-5p could sensitize PDAC cells to the chemotherapeutic efficacy of GEM by directly targeting TRAF6.

### MiR-146a-5p negatively regulates GEM resistance through the TRAF6/NF-κB p65/P-gp axis in PDAC cells

**Figure 6:**
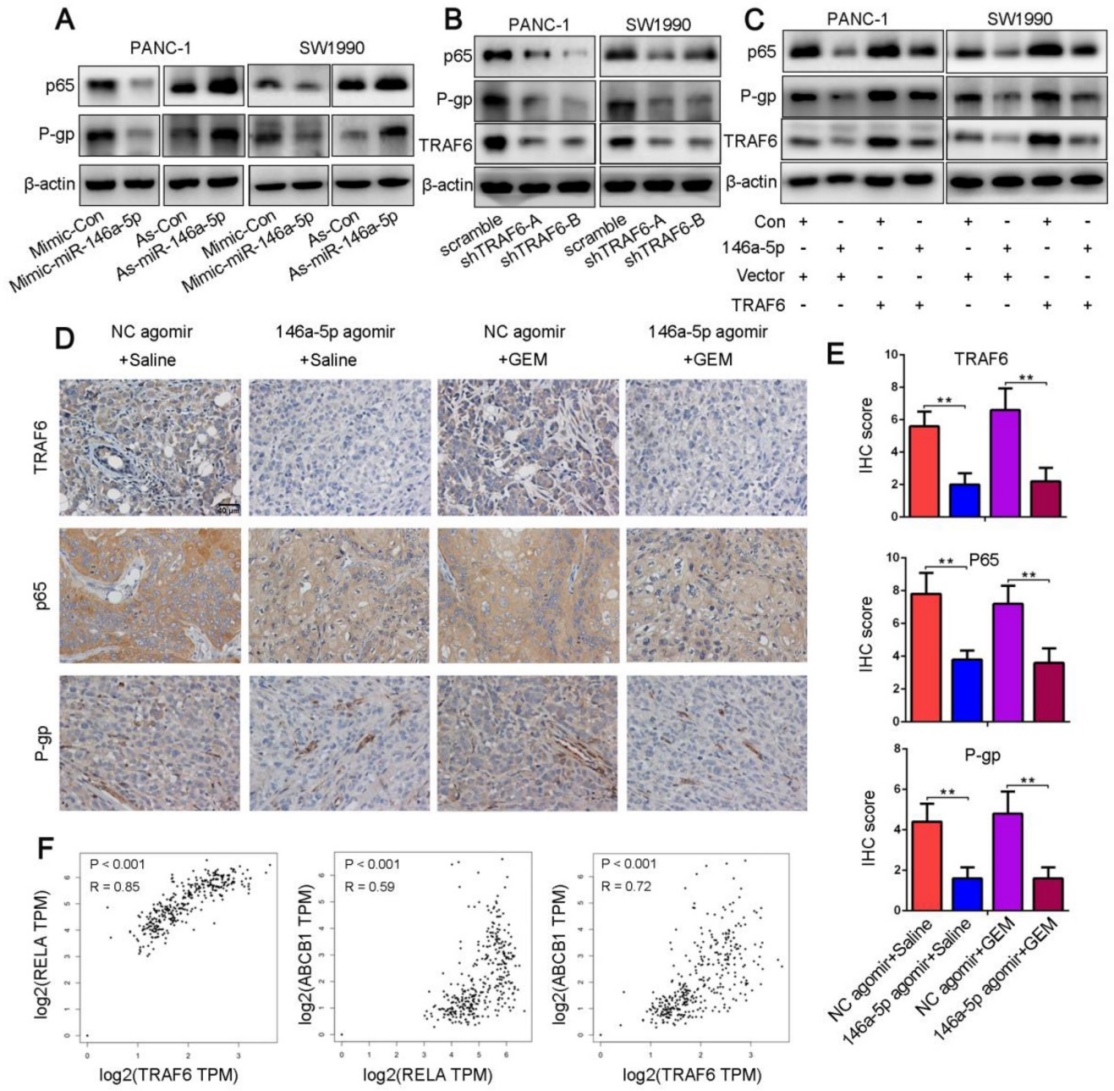
MiR-146a-5p negatively regulates GEM resistance through the TRAF6/NF-κB p65/P-gp axis in PDAC cells. (A) Western blot analysis of NF-κB p65 and P-gp levels in PANC-1 and SW1990 cells transfected with miR-146a-5p mimics, inhibitors or their NCs. (B) Western blot analysis of TRAF6, NF-κB p65 and P-gp levels in PANC-1 and SW1990 cells transfected with TRAF6 knockdown or overexpression constructs. (C) The increased NF-κB p65 and P-gp levels mediated by miR-146a-5p overexpression could be ameliorated by upregulating TRAF6, as shown by Western blot analysis. (D-E) The expression levels of TRAF6, NF-κB p65 and P-gp in the xenograft tumors as measured by IHC assays and quantified by the IHC score (scale bar, 40 µm, n = 5). (F) Both the TCGA and GTEx databases showing that TRAF6 expression was positively correlated with the expression of both NF-κB p65 (*RELA*) and P-gp (*ABCB1*) based on a Spearman correlation test.

Numerous studies have shown that NF-κB signaling is constitutively active in PDAC cells and that the increased levels of the NF-κB p65 subunit were correlated with the growth, angiogenesis, metastasis, GEM resistance and poor prognosis of PDAC 19, 22, 35-39. P-gp was identified to contribute to clinically relevant multidrug resistance and was regulated by the NF-κB signaling pathway via its binding affinity for the *ABCB1* promoter regions 25. Moreover, TRAF6 could function as a molecular bridge and key activator of NF-κB. Hence, we hypothesized that miR-146a-5p regulated the TRAF6/NF-κB p65/P-gp axis during the process of acquired GEM resistance in PDAC cells. As determined in Figure 6A, miR-146a-5p overexpression decreased the NF-κB p65 and P-gp expression levels, whereas the levels of these proteins were markedly increased in cells upon miR-146a-5p inhibition. Similarly, TRAF6 knockdown downregulated the expression levels of NF-κB p65 and P-gp (Figure 6B). In addition, the increased levels of these proteins by miR-146a-5p overexpression could be ameliorated by upregulation of TRAF6 (Figure 6C). To further confirm the above results, we examined the expression levels of TRAF6, NF-κB p65 and P-gp in xenograft tumors by IHC assays. The results revealed that the levels of these proteins were decreased in the miR-146a-5p agomir group compared with those in the NC agomir group (Figure 6D-E). Moreover, both the TCGA and GTEx databases showed that TRAF6 expression was positively correlated with the expression of NF-κB p65 (*RELA*) and P-gp (*ABCB1*) when assessed with a Spearman correlation test (Figure 6F). These results indicate that miR-146a-5p regulates GEM resistance through the TRAF6/NF-κB p65/P-gp axis in PDAC cells.

## Discussion

Chemoresistance, including de novo (intrinsic) and acquired (extrinsic) resistance, contributes to the devastating prognosis of PDAC patients 40. Hence, elucidating the mechanisms underlying chemoresistance is critical for PDAC treatment. In the present study, we constructed a PDAC-GR cell model that mimics the process of acquired chemoresistance and identified resistance-related miRNA signatures using a miRNA microarray to compare parental versus GR cell lines. After performing a series of experiments, we found that miR-146a-5p was downregulated in GR cells and that miR-146a-5p inhibited PDAC cell proliferation and resistance to GEM *in vitro* and *in vivo*. In addition, we also demonstrated that decreased miR-146a-5p expression is significantly associated with poor prognosis in PDAC patients. Collectively, our study provides a promising treatment target for modulating PDAC chemosensitivity.

Dysregulated expression of miRNAs is involved in the regulation of genes related to various kinds of diseases, including autoimmune disorders, cardiovascular disease, and many tumors 41-43. MiR-146a-5p, which under the previous nomenclature was called miR-146a, has been reported to be involved in immune function, inflammation, and individual cancers 44-46. In particular, miR-146a-5p has been labeled as a tumor promoter (oncomiRs) or tumor suppressor in numerous cancer types based on the nature of its target gene. Moreover, recent studies have suggested that miR-146a-5p also exerts either a positive or negative effect on drug resistance 47,48. Downregulated miR-146a-5p regulated imatinib mesylate resistance in human chronic myelogenous leukemia patients 48. Tan et al. reported that miR-146a plays an oncogenic role in targeting DNA damage-inducible transcript 3 and promotes non-small-cell lung cancer (NSCLC) resistance to cisplatin 47. However, whether miR-146a-5p might be involved in the chemosensitivity of PDAC has not been elucidated. In this study, we observed that downregulation of miR-146a-5p induced GEM resistance by regulating the TRAF6/NF-κB/P-gp pathway in PDAC cells. Similar to the mechanism we tested, Chen et al. found in a rat model of chronic refractory epilepsy that silencing miR-146a can improve drug resistance by regulating the HMGB1/TLR4/NF-κB/P-gp axis 49.

**Figure 7:**
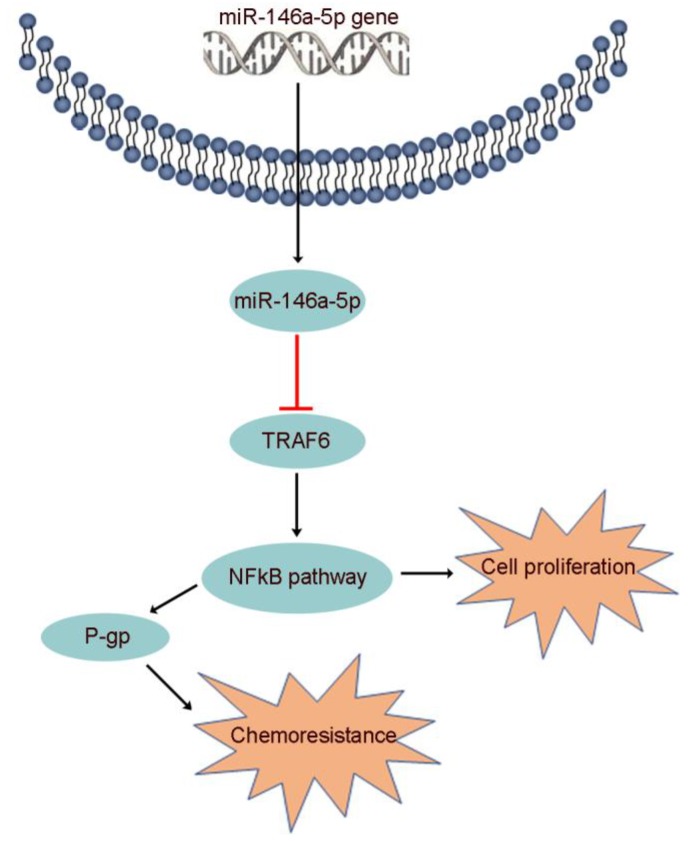
The proposed mechanism by which miR-146a-5p regulates PDAC cell growth and GEM resistance.

Increasing evidence suggests that miR-146a-5p, which binds to various downstream targets including IRAK1, EGFR, NOTCH1/2, SOX2/5 and CHOP, plays a role in many biological processes 44. However, the function of miR-146a-5p and the association of its target gene with GEM resistance in PDAC were unclear. Here, we identified TRAF6 as a potential target of miR-146a-5p through bioinformatics analysis. Moreover, recent studies have indicated that TRAF6 regulates tumorigenesis in various cancers, including PDAC 26,27. Our data identified the oncogenic role of TRAF6 in increasing GEM resistance and its correlation with poor prognosis in PDAC. P-gp, also known as multidrug resistance 1 (MDR1), plays an important role in the efflux of many drugs, including GEM, and enhanced chemoresistance, the latter of which is regulated by activation of the NF-κB signaling pathway 25,50. Subsequently, we further determined that miR-146a-5p regulated the TRAF6/NF-κB p65/P-gp axis in the process of GEM resistance in PDAC cells (Figure 7).

Several studies have examined the impact of regulatory proteins on miR-146a-5p expression in cancer cells. Kumaraswamy et al. reported that BRCA1-mediated upregulation of miR-146a by binding to its promoter leads to the repression of direct miR-146a targets in breast cancer cell lines 51. Similarly, STAT3, a transcriptional corepressor, can bind to the miR-146a promoter and induce its expression in hepatocellular carcinoma cells 52. Furthermore, epigenetic mechanisms, including DNA CpG methylation, histone acetylation, and histone methylation, contribute to the dysregulation of miR-146a expression in cancer 44. Iacona et al. reported that DNA methylation at CpG sites in promoter regions contributes to decreased miR-146a expression in NSCLC cells 53. Therefore, the regulation of miR-146a-5p in cancer is a complex process that requires further exploration.

Numerous studies have demonstrated that miRNAs have been used as biomarkers to facilitate early diagnosis and prognosis and to predict treatment response 54. We found that miR-146a-5p dysregulation in PDAC tissues were correlated with OS of patients. Moreover, our data indicate that miR-146a-5p expression is a potential biomarker for chemotherapy selection in patients with PDAC who receive GEM-based or fluoropyrimidine-based chemotherapy. Similarly, NSCLC patients with increased serum levels of miR-146a had a better response to chemotherapy and experienced better OS than patients with lower levels, as reported previously by Wu et al. 55. Hence, additional studies are required to confirm that miR-146a-5p expression is a potential biomarker for chemotherapy selection in patients with PDAC.

Taken together, the data from our study provide a new understanding of miR-146a-5p in PDAC cell growth and GEM resistance through the regulation of TRAF6/NF-κB signaling, which may represent a novel regulatory mechanism for PDAC. Hence, biological or pharmacological intervention targeting miR-146a-5p may be a promising strategy to improve chemotherapeutic efficacy. Furthermore, the expression levels of miR-146a-5p may have prognostic significance and act as a vital predictive biomarker for clinical chemotherapy selection in PDAC patients.

## Supplementary Material

Supplementary figures and tables.

## Abbreviations

miRNA: microRNA
GEM: gemcitabine
PDAC: pancreatic ductal adenocarcinoma
TRAF6: tumor necrosis factor receptor-associated factor 6
ATCC: American Type Culture Collection
HPDE: Human pancreatic ductal epithelial
GR cells: GEM-resistant cells
TMAs: Tissue microarrays
FUSCC: Fudan University Shanghai Cancer Center
qRT-PCR: quantitative real-time PCR
P-gp: P-glycoprotein
IHC: Immunohistochemical
TCGA: The Cancer Genome Atlas
PA cells: parental cells
IC50: inhibitory concentration
GEO: gene expression omnibus
OS: overall survival
DFS: disease-free survival
WT: wild-type
MU: mutated
GTEx: genotype-tissue expression project
NSCLC: non-small-cell lung cancer
MDR1: multidrug resistance 1
