# Supplementary material for: A miR-146a-5p/TRAF6/NF-kB p65 axis regulates pancreatic cancer chemoresistance: functional validation and clinical significance
Source: Theranostics. 2020 Mar 4;10(9):3967–79. doi: 10.7150/thno.40566 (PMC7086345; doi:10.7150/thno.40566)
Supplement: Supplementary file 1 — Supplementary figures and tables. [file thnov10p3967s1.pdf]

Supplementary figure legends

Figure S1. PDAC cell line models (MiaPaCa-2-GR and SW1990-GR cells) with acquired GEM resistance were constructed. (A) The parental cells (PA cells) were cultured with increasing GEM doses over a period of approximately 6 months. (B-C) The inhibitory concentration (IC_{50}) of GEM of the resistant cells was determined by using CCK-8 assays, and the GR cells were more resistant than the PA cells ($P < 0.05$). (D) Our qRT-PCR data showed low miR-146a-5p expression in the GR cell lines compared with the expression in the corresponding PA cells.

Figure S2. MiR-146a-5p sensitizes the chemotherapeutic efficacy of SW1990 cells *in vivo*. (A) Representative images showing the tumors formed in the four groups (n = 5 for each group). (B) Tumor growth curves were drawn according to the tumor volumes measured (**$P < 0.01$, ***$P < 0.001$). (C) Tumor weights of the four groups were measured at the 5th week after subcutaneous transplantation (**$P < 0.01$, ***$P < 0.001$). (D-E) Representative tumor tissue sections of the xenografts from four groups were analyzed for the proliferation marker Ki-67 using IHC, and the percentages of Ki67-positive cells were measured (scale bar, 40 μm, **$P < 0.01$).

Figure S3. The increased TRAF6 in PDAC tissues is associated with clinicopathological features. (A) TCGA and genotype-tissue expression project (GTEx) databases also showed increased TRAF6 in PDAC tissues compared with that in normal tissues. (B) Representative images of high and low expression of TRAF6 in TMA analysis by IHC staining. (C) There was obvious relationship between TRAF6 expression and clinicopathological features, such as differentiation, positive lymph nodes, and TNM stage. Statistical significance was determined by the χ^2-test.

Figure S1

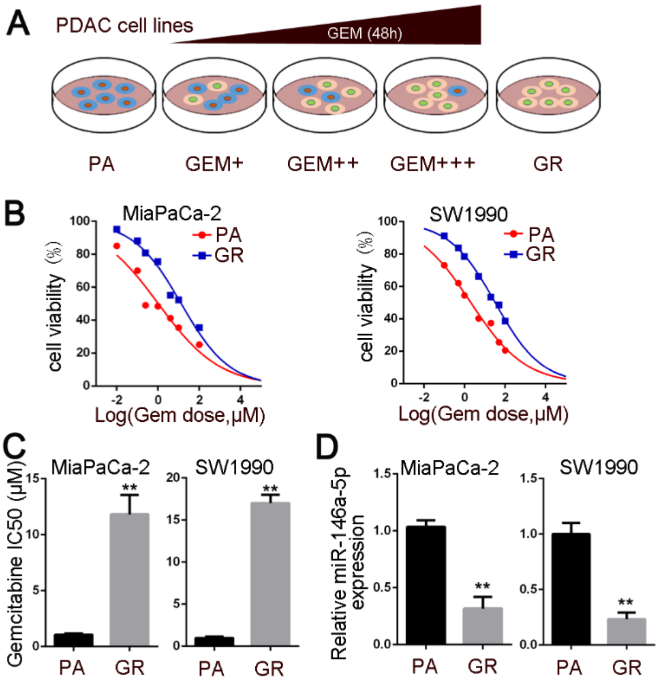
A
PDAC cell lines
GEM (48h)
PA
GEM+
GEM++
GEM+++
GR
B
MiaPaCa-2
SW1990
PA
GR
cell viability (%)
Log(Gem dose,μM)
C
Gemcitabine IC50 (μM)
**
D
Relative miR-146a-5p expression

Figure S2

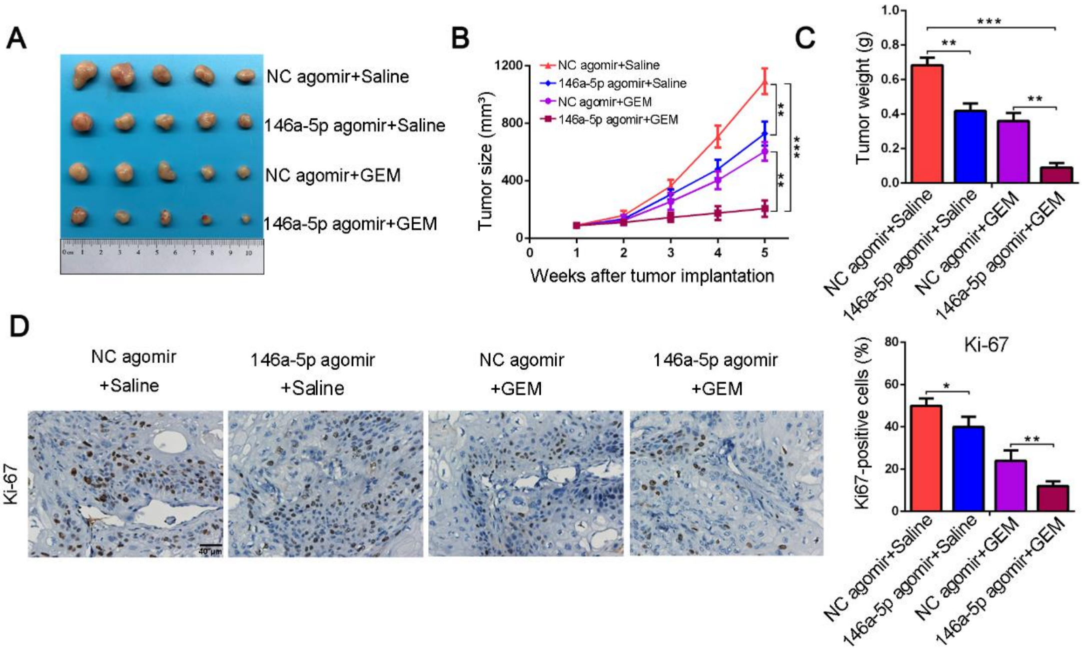
A
NC agomir+Saline
146a-5p agomir+Saline
NC agomir+GEM
146a-5p agomir+GEM
B
NC agomir+Saline
146a-5p agomir+Saline
NC agomir+GEM
146a-5p agomir+GEM
Tumor size (mm³)
Weeks after tumor implantation
C
Tumor weight (g)
D
NC agomir +Saline
146a-5p agomir +Saline
NC agomir +GEM
146a-5p agomir +GEM
Ki-67
Ki67-positive cells (%)

Figure S3

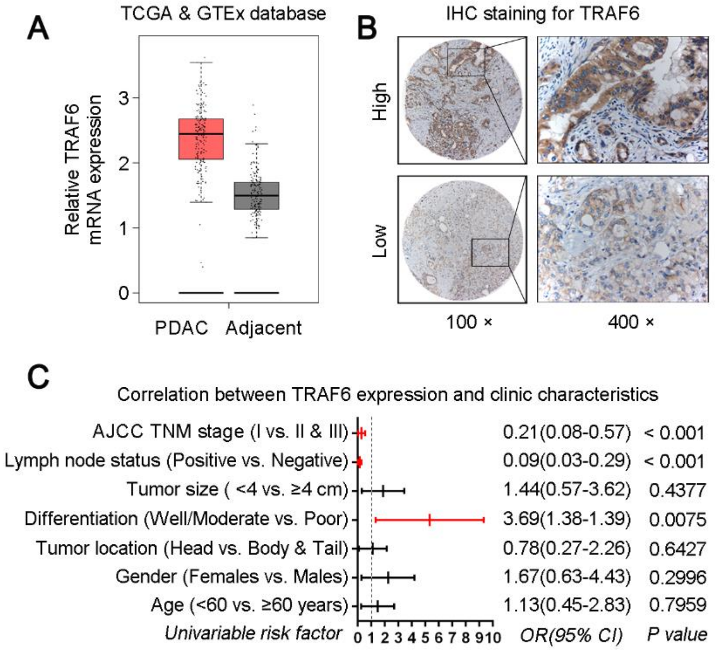

A
TCGA & GTEx database
Relative TRAF6 mRNA expression
0
1
2
3
PDAC
Adjacent
B
IHC staining for TRAF6
High
Low
100 ×
400 ×
C
Correlation between TRAF6 expression and clinic characteristics
AJCC TNM stage (I vs. II & III)
Lymph node status (Positive vs. Negative)
Tumor size (<4 vs. ≥4 cm)
Differentiation (Well/Moderate vs. Poor)
Tumor location (Head vs. Body & Tail)
Gender (Females vs. Males)
Age (<60 vs. ≥60 years)
Univariable risk factor
0 1 2 3 4 5 6 7 8 9 10
OR(95% CI)
P value
0.21(0.08-0.57) < 0.001
0.09(0.03-0.29) < 0.001
1.44(0.57-3.62) 0.4377
3.69(1.38-1.39) 0.0075
0.78(0.27-2.26) 0.6427
1.67(0.63-4.43) 0.2996
1.13(0.45-2.83) 0.7959

Table S1. Primer sequences used in the study.

**Genes (*Homo sapiens*)**	**Primers**	**Sequences**
miR-146a-5p	Forward	5'-CGCGTGAGAACTGAATTCCA-3
	(Stem-loop)	5'-AGTGCAGGGTCCGAGGTATT-3
TRAF6	Forward	5'-GCCCATGCCGTATGAAGAGA-3'
	Reverse	5'-ACTGAATGTGCAGGGGACTG-3’
β-actin	Forward	5'-CTACGTCGCCCTGGACTTCGAGC-3'
	Reverse	5'-GATGGAGCCGCCGATCCACACGG-3'

Table S2. Clinicopathological features and correlation of miR-146a-5p expression in PDAC tissue samples.

Characteristics	No. of patients (n=93)	miR-146a-5p Low expression (n=60)	miR-146a-5p High expression (n=33)	*P* value
**Age**				
<60 years, n (%)	39(41.93)	25(41.66)	14(42.42)	0.9435
≥60 years, n (%)	54(58.07)	35(58.34)	19(57.58)	
**Gender**				
Female, n (%)	45(48.39)	28(46.67)	17(51.52)	0.6544
Male, n (%)	48(51.61)	32(53.33)	16(48.48)	
**Tumor location**				
Head, n (%)	58(62.36)	33(55.00)	24(72.73)	0.0931
Body and Tail, n (%)	35(37.64)	27(45.00)	9(27.27)	
**Tumor size**				
＜4.0 cm, n (%)	69(74.19)	45(75.00)	24(72.73)	0.8106
≥4.0 cm, n (%)	24(25.81)	15(25.00)	9(27.27)	
**Lymph node status**				
Negative, n (%)	56(60.22)	34(56.67)	22(66.67)	0.3458
Positive, n (%)	37(39.78)	26(43.33)	11(33.33)	
**Tumor differentiation**				
Well/Moderate, n (%)	56(60.22)	36(60.00)	20(60.61)	0.9544
Poor, n (%)	37(39.78)	24(40.00)	13(39.39)	
**TNM Stage**				
I, n (%)	33(35.48)	19(31.67)	14(42.42)	0.2995
II and III, n (%)	60(64.52)	41(68.33)	19(57.58)	

Table S3. Univariate and multivariate Cox regression of overall survival for patients with PDAC.

Characteristics	Univariate			Multivariate		
	HR	95% CI	*P* value	HR	95% CI	*P* value
**Age**						
< 60 years<br>≥ 60 years	0.75	0.47 to 1.19	0.1098			
**Gender**						
Female<br>Male	1.09	0.69 to 1.73	0.7089			
**Tumor location**						
Head<br>Body and Tail	1.18	0.75 to 1.87	0.4795			
**Tumor size**						
≥4.0 cm<br><4.0 cm	0.43	0.24 to 0.78	0.0126	2.03	1.23 to 3.35	0.0060
**Tumor differentiation**						
Well/Moderate<br>Poor	0.80	0.49 to 1.29	0.1327			
**Lymph node status**						
Positive<br>Negative	0.35	0.20 to 0.60	< 0.001			
**TNM Stage**						
I<br>II and III	0.38	0.23 to 0.61	< 0.001	2.43	1.51 to 3.93	< 0.001
**miR-146a-5p expression**						
Low<br>High	0.57	0.36 to 0.90	0.0185	0.54	0.33 to 0.88	0.0140

Table S4. The potential targets of microRNA-146a-5p

TargetScan	miRanda	PicTar	RNA22	PITA
IGSF1	NOVA1	PPP1R11	IRAK1	TRAF6
KBTBD4	RHOXF2B	NFAT5	TRAF6	SRSF6
PSMA4	RHOXF2	PAPPA	NOTCH1/2	SEC23IP
CDKN2AIP	TPM1	ZDHHC17	EGFR	NOVA1
ZBTB2	TRAF6	SORT1	RPA3	WWC2
IRAK1	ZNF826	RNF31	CHOP	UPP2
SLC10A3	IGSF1	CNTNAP2	BRCA1	PPP1R11
HNRNPD	PCGF5	NF2	ALOX5AP	BCORL1
ZDHHC13	ZNF253	ELAVL1	SMAD4	SORT1
NOVA1	ZNF90	HIPK1	ST8SIA4	ZNF649
TRAF6	LIN52	GAD1	LIN52	PLSCR4
TDRKH	TMEM185B	SON	ATG12	NUMB
AC012215.1	ITCH	PIP5K2B	CXCR4	USP32
TMEM120B	PHC1	STC1	RHOA	APPL1
RARB	ABCD3	EIF5A2	SOD2	ZNF652
LFNG	CMAH	ZAK	RAC1	SIAH2
CD80	LRRTM3	PTGFRN	HAb18G	IRAK1
ACKR2	PMS1	HSHIN1	PTGS2	CARD10
NUMB	TFAP2D	PRX	L1CAM	GDAP1L1
KLF7	LRCH1	SDFR1	COPS8	EIF4G2
NUDT17	MGC11082	EFNB2	CARD10	GRIA3
NRAS	TMEM19	TRAF6	PRKCE	MRS2
EIF4G2	ADRB2	NOVA1	UHRF1	ZNF354B
ZSCAN29	RASGRP1	MXD4	RARB	SLC10A3
USP3	FBXL3	ABCC12	CCNJ	ZNF540
AFAP1L2	LCA5L	SYNPR	LIN52	TDRKH
TMEM194A	UBE2J1	SH3GL2	EGR1	DDHD1
ERLEC1	RAB7L1	RAB10	ROCK	BRK1
FAM169A	UHRF1	IRAK1	TAK1	LOC388813
FAM26E	SLC16A14	KIAA0141	WASF2	POFUT2

Table S5. Clinicopathological features and correlation of TRAF6 expression in PDAC tissue samples.

Characteristics	No. of patients (n=87)	TRAF6 High expression (n=61)	TRAF6 Low expression (n=26)	*P* value
**Age**				
<60 years, n (%)	42(48.28)	30(49.18)	12(46.15)	0.7959
≥60 years, n (%)	45(51.72)	31(50.82)	14(53.85)	
**Gender**				
Female, n (%)	34(39.08)	26(42.62)	8(30.77)	0.2996
Male, n (%)	53(60.92)	35(57.38)	18(69.23)	
**Tumor location**				
Head, n (%)	64(73.56)	44(72.13)	20(76.92)	0.6427
Body and Tail, n (%)	23(26.44)	17(27.87)	6(23.08)	
**Tumor size**				
＜4.0 cm, n (%)	49(56.32)	36(59.02)	13(50.00)	0.4377
≥4.0 cm, n (%)	38(43.68)	25(40.98)	13(50.00)	
**Lymph node status**				
Negative, n (%)	22(25.28)	7(11.48)	15(57.69)	< 0.001
Positive, n (%)	65(74.71)	54(88.52)	11(42.31)	
**Tumor differentiation**				
Well/Moderate, n (%)	61(70.11)	48(78.69)	13(50.00)	0.0075
Poor, n (%)	26(29.89)	13(21.31)	13(50.00)	
**TNM Stage**				
I, n (%)	26(29.89)	12(19.67)	14(53.85)	< 0.001
II and III, n (%)	61(70.11)	49(80.33)	12(46.15)	
